# Influence of G protein-biased agonists of μ-opioid receptor on addiction-related behaviors

**DOI:** 10.1007/s43440-021-00251-1

**Published:** 2021-04-09

**Authors:** Lucja Kudla, Ryszard Przewlocki

**Affiliations:** Department of Molecular Neuropharmacology, Maj Institute of Pharmacology Polish Academy of Sciences, ul. Smetna 12, 31-343 Krakow, Poland

**Keywords:** G protein-biased opioids, Μ-opioid receptor, Addiction, Tolerance, Reward, Dependence

## Abstract

Opioid analgesics remain a gold standard for the treatment of moderate to severe pain. However, their clinical utility is seriously limited by a range of adverse effects. Among them, their high-addictive potential appears as very important, especially in the context of the opioid epidemic. Therefore, the development of safer opioid analgesics with low abuse potential appears as a challenging problem for opioid research. Among the last few decades, different approaches to the discovery of novel opioid drugs have been assessed. One of the most promising is the development of G protein-biased opioid agonists, which can activate only selected intracellular signaling pathways. To date, discoveries of several biased agonists acting via μ-opioid receptor were reported. According to the experimental data, such ligands may be devoid of at least some of the opioid side effects, such as respiratory depression or constipation. Nevertheless, most data regarding the addictive properties of biased μ-opioid receptor agonists are inconsistent. A global problem connected with opioid abuse also requires the search for effective pharmacotherapy for opioid addiction, which is another potential application of biased compounds. This review discusses the state-of-the-art on addictive properties of G protein-biased μ-opioid receptor agonists as well as we analyze whether these compounds can diminish any symptoms of opioid addiction. Finally, we provide a critical view on recent data connected with biased signaling and its implications to in vivo manifestations of addiction.

**Graphic abstract:**

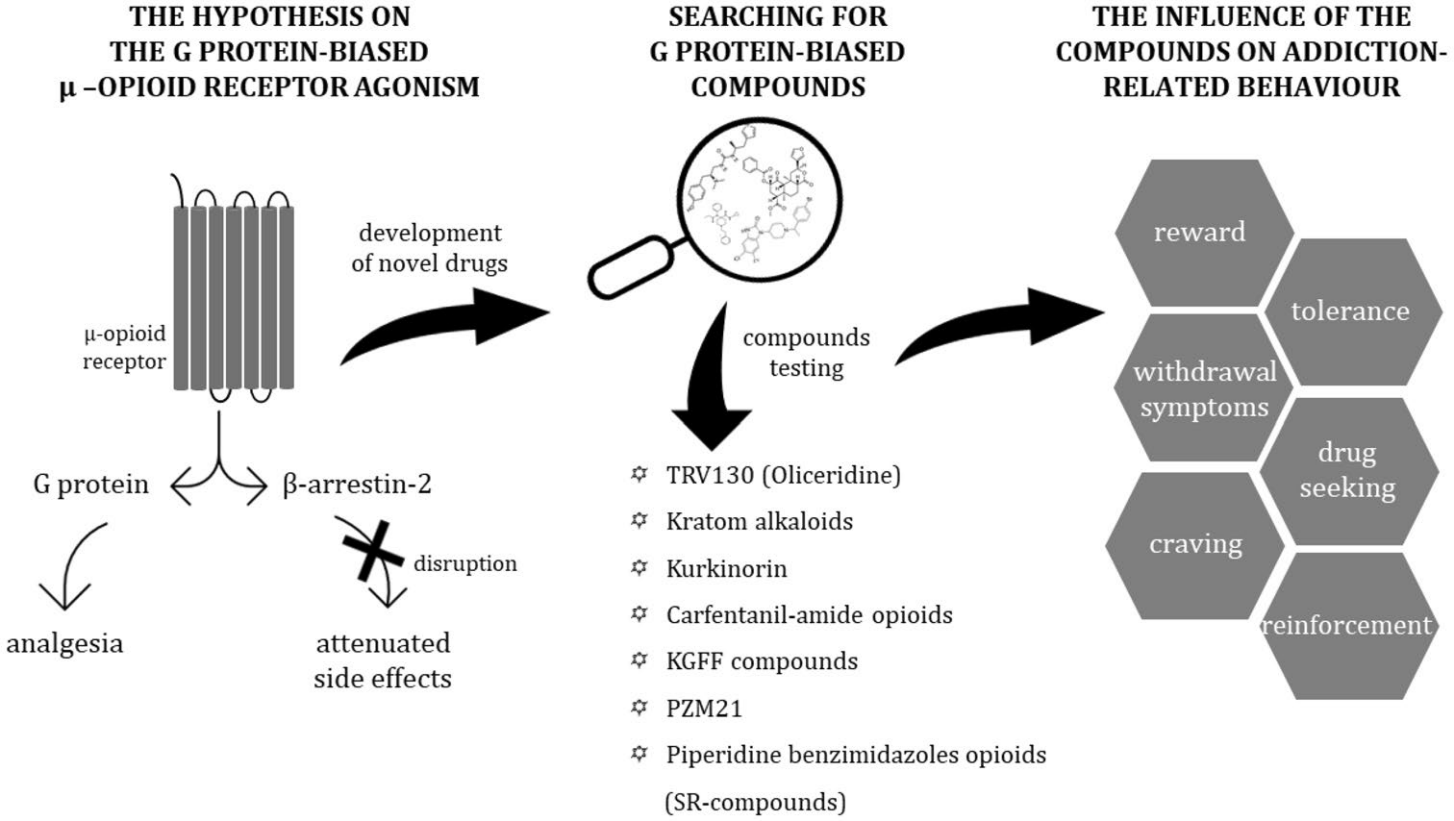

## Introduction

The primary goal of recent opioid research is to design and synthesize novel potent analgesic agents devoid of adverse side effects. Among a body of undesirable effects of conventional opioid drugs, their high addictive potential appears as extremely important [1], especially in the context of the opioid overdose crisis, which currently is a serious health, social, and economic problem [2, 3].

Opioid receptors, including μ-opioid receptor (μ-OR), belong to the family of G protein-coupled receptors (GPCRs), which are a large group of proteins that are cell surface receptors binding extracellular substances and transmitting intracellular signals via a G protein [4]. At the cellular level, an agonist binding to the μ-OR activates G_i/o_ proteins and, as a result, leads to a number of intracellular events, including the inhibition of adenylate cyclase and modulation of the activity of certain ion channels. Although an acute stimulation of the μ-OR results in a decrease in cyclic AMP (cAMP) level, its prolonged exposition on the agonist leads to an increase in cAMP levels which is a molecular marker of opioid tolerance and dependence [5]. μ-OR signaling is determined by several regulatory mechanisms, such as receptor desensitization and internalization. A prominent role in these mechanisms has been attributed to β-arrestin2 (β-arr2) which is a scaffolding protein binding to the phosphorylated receptor. This intracellular protein plays a crucial role in mediating the internalization of the receptor, as the binding of an activated and phosphorylated receptor with β-arr2 allows receptor internalization resulting in fewer receptors available for further activation by an agonist. These mechanisms were proposed to underlie the reduced agonist signaling after chronic agonist exposure [6, 7]. Importantly, binding of β-arr2 is a crucial step for redirection of the μ-OR signaling to the alternative pathway [8–11]. Thus, upon the μ-OR stimulation, two independent signaling pathways, G protein- and β-arr2-dependent, are activated.

Research of the last 2 decades has brought interesting discoveries in the area of the group of G protein-biased (also known as functionally selective) opioid agonists [12]. Biased agonism is a term that refers to an agonist’s ability to preferentially signal via one intracellular pathway over another. It is the ligand-dependent selectivity for the certain intracellular signaling pathway relative to the reference agonist of the same receptor [13]. This concept has been introduced in the opioid field as a mean to split desirable and adverse effects of opioid therapeutics. The starting point for the development of this field of opioid pharmacology were research on the functional role of β-arr2 in behavioral responses to morphine. Studies in mice lacking β-arr2 have shown that β-arr2-dependent signaling pathway may be responsible for at least some of opioid side effects [14, 15]. It resulted in a novel pharmacological approach which is focused on the attempts to the development of compounds able to preferentially activate a G protein pathway with minimal engagement of β-arr2. Therefore, the hypothesis assumes that analgesic and adverse effects of μ-OR activation may be separated into biased ligands. In particular, μ-OR agonists biased to G protein, but not to β-arr2, could be effective analgesics devoid of side effects, including opioid abuse.

**Fig. 1:**
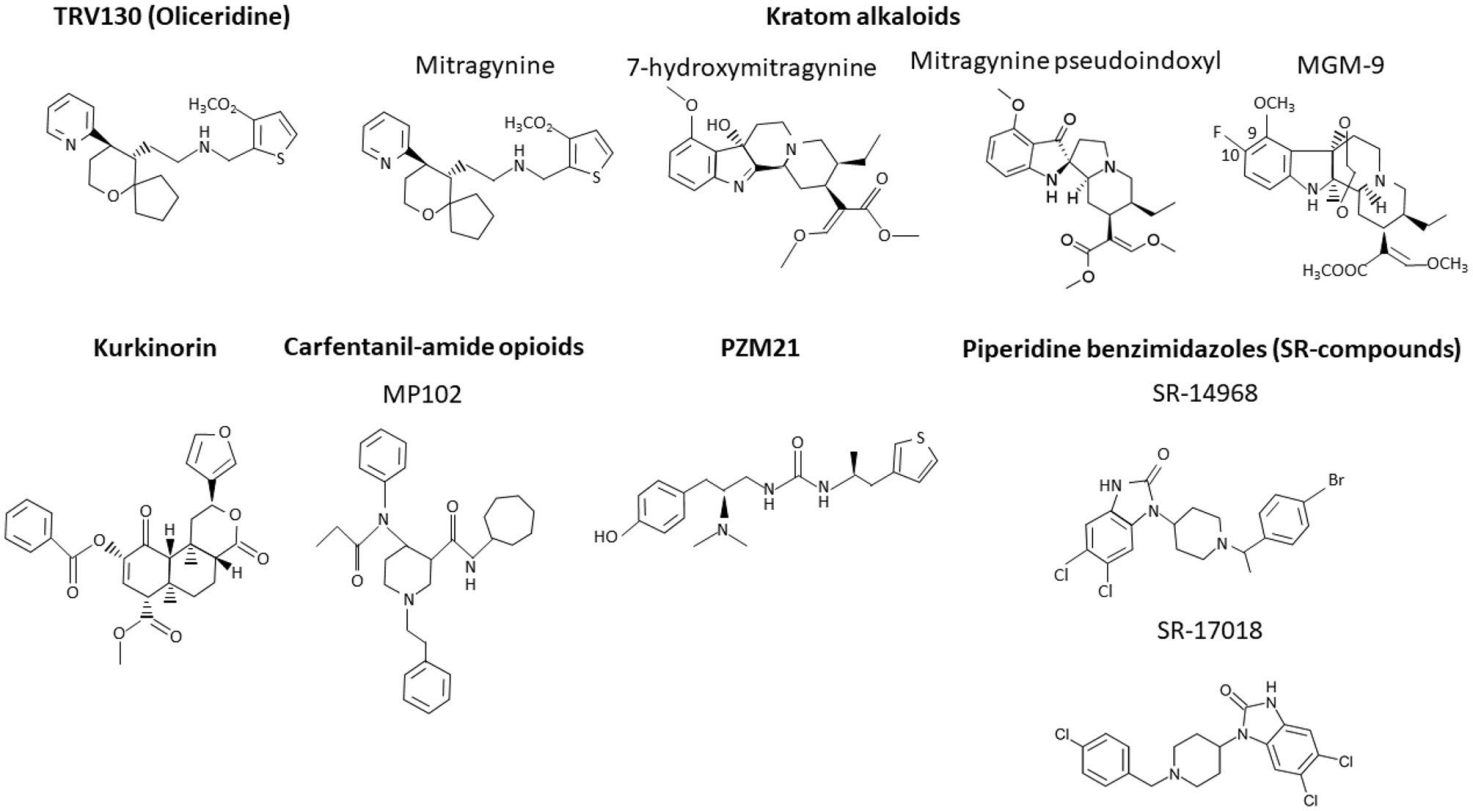
Chemical structures of discussed ligands. The figure presents chemical structures of agonists in the order they are discussed in the article: TRV130 (Oliceridine), kratom alkaloids, kurkinorin, carfentanil-amide opioids (MP102 as an exemplary member of this family of compounds), PZM21 and piperidine benzimidazoles (SR-compounds; SR-14968 and SR-17018 were chosen and discussed as the most interesting and promising compounds from this group)

Following this idea, several G protein-biased μ-OR agonists (Fig. 1) have been discovered; however, their effects are incompletely explored. If their addictive potential is considered, there is no consensus on this matter, as different studies have reached mixed results. Interestingly, some of the compounds were also assessed in terms of their effects on symptoms of addiction to other, unbiased opioids and this research field is an interesting alternative to the use of new ligands.

In this review, we present and critically assess the state-of-the-art on the addictive potential of most promising G protein-biased μ-OR agonists and their influence on behavioral aspects of opioid addiction. Based on the available data and theoretical knowledge regarding the relationship between G protein/β-arr2-dependent pathways and desired and side effects of opioid drugs, we discuss the possible influence of biased signaling, but also alternative explanations, on in vivo effects of the reviewed compounds. Although several well-written and comprehensive reviews discussing the biased signaling at opioid receptors have been recently published [16–18], here we aimed to put particular emphasis on behavioral studies of the most interesting compounds in the context of their addictive and potentially antiaddictive properties. As the problem of the opioid overdose crisis is a major challenge for public health, our question is whether and how can we face this problem using G protein-biased opioids.

## Opioid epidemic

Opioid drugs have been misused for many years; however, over the past few decades, their use has increased dramatically, leading to a worldwide opioid epidemic. Statistics provided by the International Narcotics Control Board indicate that about 53 million people (1.1% of the general population between 15 and 64 years old) abused opioids in 2019 [19]. From 1999 to 2017, a sixfold increase in deaths associated with opioid misuse was noticed, what emphasizes the rapid development of the crisis [20]. Only in Europe, 80–90% of the total number of 9 400 drug overdose deaths were attributed to opioids (in 2017; [19]). Importantly, many of users intake synthetic opioids for nonmedical causes, which presumably hugely contribute to statistics; however specific numbers are not available. The crisis has broken out in the United States and Canada and opioid overuse-related death is the most lethal drug epidemic in the history of America [21]. Therefore, substantial efforts of medical and scientific societies are aimed to reduce the health, social, and economic effects of the epidemic [22–24]. Among different scientific approaches focused on the containment of the crisis, several have gained exceptional attention [25]. The goal of all of them is to discover novel compounds devoid of undesirable effects, including addiction.

## Opioid use disorder (OUD)

Opioids are one of the most often misused drugs of abuse and addiction to them is usually associated with much greater harm than to other narcotics [26]. Persistent exposure to opioid drugs leads to adaptations in brain function that induce behavioral and physiological symptoms of drug dependence and addiction [1]. According to DSM-5, OUD is a chronic disorder characterized by a problematic pattern of opioid use resulting in problems or distress [27]. An addicted person continues to use opioid despite having recurring social or interpersonal problems and not fulfilling obligations in personal and professional life. Most individuals begin to use opioid substances to feel a characteristic “high” or to diminish pain. However, through desensitization of receptors, repeated opioids use results in tolerance development, which in turn often causes their uncontrolled intake. Typical time course of this disorder includes an initial period of recreational use when rewarding and analgesic properties of opioids dominate; then, due to tolerance, a person is forced to take higher and higher doses of a drug to obtain the same effect and craving appears; finally, withdrawal periods are associated with serious physiological (muscle and bone pain, tearing, diarrhea, and abdominal cramps) and psychological symptoms (agitation, anxiety), which usually results in drug reuse to alleviate these undesirable feelings [21, 28].

To study basic mechanisms underlying OUD and to test novel compounds, animal models are commonly used. As OUD is a complex and multidimensional condition, it is difficult to mimic all of its symptoms in these models. In human, it is a chronic disease with the varying course, while animal tests, such as conditioned place preference or drug-self-administration, allow exploring only selected aspects of addictive behaviors, such as euphoric, rewarding, or reinforcing effects of opioid drugs. However, several well-established behavioral assays allow us to investigate the most prominent addiction symptoms in laboratory rodents [29]. Verified and replicable models are useful tools for the assessment of both physiological (related to dependence processes, i.e., tolerance and withdrawal symptoms) and subjective (connected to motivation and reward processing) aspects of opioid addiction [29, 30]. Tests used for studying addictive effects of G protein-biased opioids discussed in this work are presented in Table 1. Efforts aimed at limiting all negative symptoms of this disease can include two alternatives: one targeted at the development of novel compounds with analgesic properties, but reduced abuse potential and second—focused on the generation of effective drugs, useful in the pharmacotherapy of OUD.

**Table 1:** Brief description of main behavioral paradigms used for studying opioid addiction symptoms

Physiological aspects of opioid addiction	
Tolerance to antinociceptive effects	Measurement of antinociceptive efficacy of a given agonist over a period of time using thermal or mechanical nociceptive tests [31]
Withdrawal syndrome	Naloxone-precipitated or spontaneous withdrawal symptoms in dependent animals measured over a period of time. Most common withdrawal symptoms include: jumping, body/paw tremor, teeth chattering, rearings, wet-dog shakes, increased urination and defecations [32]

Subjective aspects of opioid addiction	
Reward-associated behavior	Conditioned place preference paradigm allowing to measure animal’s preference toward the drug-associated area in comparison to the neutral environment [33]
Reinforcement-related behavior	Operant self-administration (intravenous, oral) of a substance under stable or progressive ratio conditions allowing to measure motivation to obtain drug [34] Relapse-related and drug-seeking behaviors during the abstinence period [35] Drug discrimination test used for identifying the abuse potential of a given compound in comparison to known drugs of abuse [36] Intracranial self-stimulation procedure which through electrical stimulation of brain reward region allows to assess the effects of various pharmacological manipulations on sensitivity to reward [37]

## Biased agonism as a novel pharmacological approach to search for safer and nonaddictive opioids

**Fig. 2:**
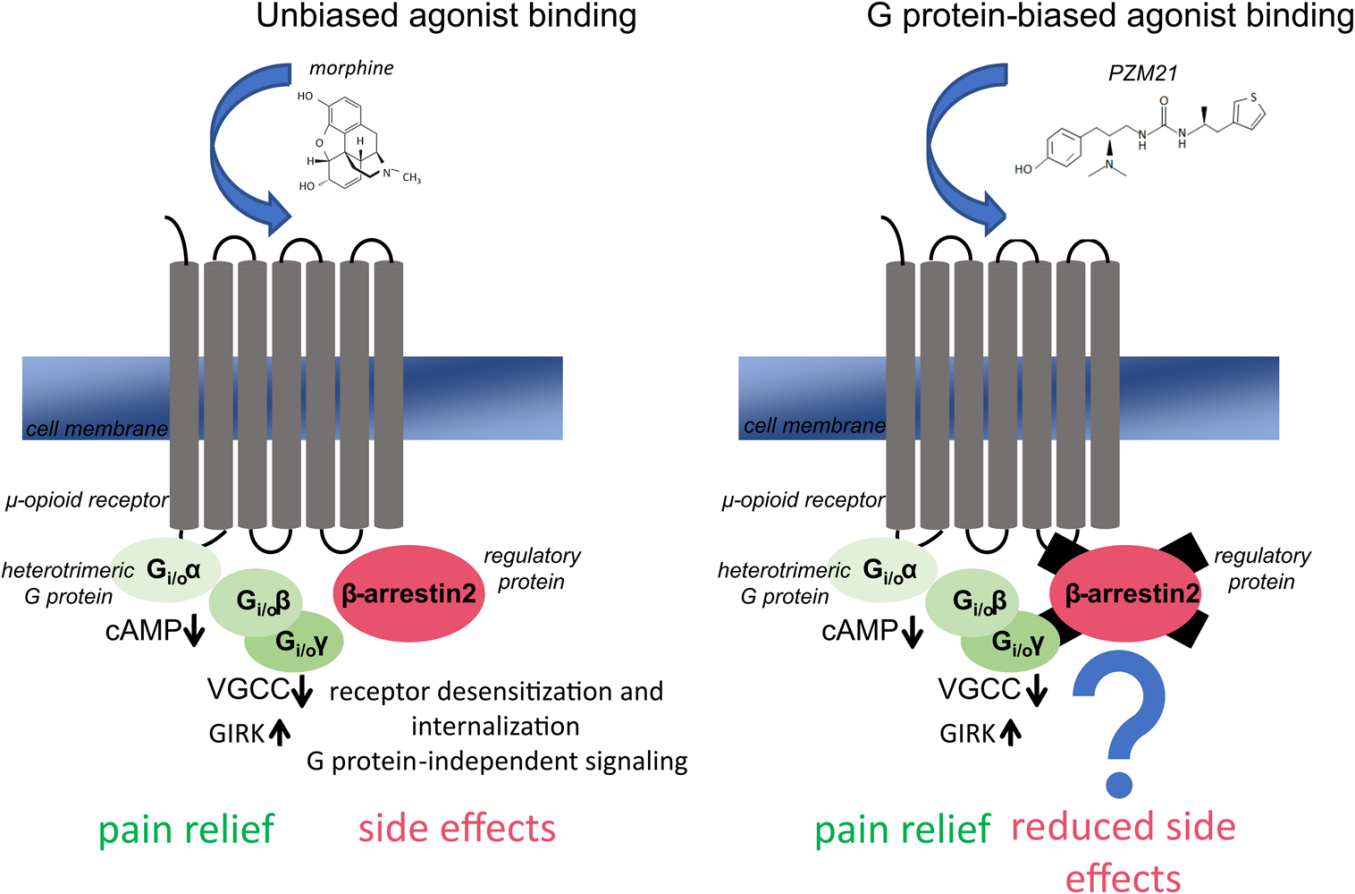
Activation of the μ-OR by unbiased and biased agonists. Following an unbiased agonist binding to the μ-OR both G_i/o_ protein- and β-arr2-dependent signaling pathways are activated. The conformation changes detach the G_i/o_α and G_i/o_βγ subunits of the heterotrimeric G protein. The G_i/o_α subunit inhibits the activity of adenylate cyclase, reduces cAMP production, and leads to the activation of downstream signaling pathways, while the G_i/o_βγ subunit inhibits voltage-gated calcium channels (VGCC) and activates G protein coupled inwardly rectifying potassium channels (GIRK) which results in hyperpolarization and inhibition of neuronal activity. This pathway is thought to mediate analgesia. On the other hand, the β-arr2-dependent pathway has been linked with undesirable opioid effects. It is hypothesized that the exclusion of this pathway using agonists with G protein bias may diminish opioid side effects, while preserving therapeutic ones

μ-OR, a member of the GPCRs family, is the main target for the action of opioid analgesics (Fig. 2). An agonist binding to this receptor leads to the conformation changes and activation of heterotrimeric G_i/o_ protein, composed of α, β, and γ subunits. The activated Gα subunit exchanges GDP into GTP which triggers the dissociation of Gα subunit from the Gβγ dimer. Then, Gα and Gβγ subunits reach the target sites, which are effector proteins, and modulate their activity [38]. The G_i/o_α subunit inhibits the adenylate cyclase, production of cAMP, and finally activates downstream signaling pathways, whereas the G_i/o_βγ subunit function is inhibition of voltage-gated calcium channels (VGCC) and activation of G protein coupled inwardly rectifying potassium channels (GIRK), leading to suppression of neuronal excitability. Hence, stimulation of μ-OR alters neuronal functions and as a result, leads to the inhibition of neuronal activity by G protein signaling [39]. The G protein pathway promotes receptor-specific effects, most commonly analgesia. Among others, it was shown that adenylate cyclase and cAMP are involved in nociception and opioid-dependent analgesia, because enhanced nociception is associated with elevated cAMP level [40]. This signaling is controlled by various kinases and regulatory proteins, including β-arr2 which is known from its control over GPCRs desensitization, internalization, and intracellular trafficking [41]. β-arr2 regulates opioid receptors signaling, as its binding facilitates receptor internalization and finally redirects signaling to alternative G protein-independent pathways [42].

The agonist stimulation of μ-OR results in the activation of G protein- and β-arr2-dependent signaling pathways [39]. Each agonist of this receptor triggers these pathways with higher or lower efficacy. Both endogenous and exogenous agonists of the μ-OR can be classified into one of the three groups: balanced agonists (activating both signaling pathways to a similar extent), G protein-biased (preferentially activating the G protein pathway) and β-arr2-biased (preferentially acting by the β-arr2 pathway). What is important, ‘bias’ is a relative term, as the bias factor is estimated according to the performance of the compound in assays measuring its activity at both signaling pathways. The higher bias factor refers to the greater difference between an agonist’s performance in the two assays in comparison to the performance of the reference ligand [43]. Therefore, bias may vary depending on the reference agonist and in vitro tests used in the study.

To date, only a few studies were aimed to assess whether endogenous opioid peptides display signaling bias. The study by Thompson et al. [44] has shown that some of the endogenous opioid peptides lacked significant bias when comparing [^35^S]GTPγS binding and β-arr2 recruitment measurements. However, Gomes et al. [45] demonstrated that some of the peptides present biased signaling at all opioid receptors. Many peptides behaved as balanced μ-OR agonists; however, some of them presented distinct biased profiles. Therefore, the studies underly the complexity of endogenous opioid signaling.

As mentioned above, the bias factor is a feature that can be differently reported across studies, depending on the reference ligand and cellular assays used. DAMGO, a highly selective synthetic μ-OR peptide agonist, is the most commonly used reference balanced ligand, meaning that it involves both signaling pathways to the similar extent. Morphine, a prototypic μ-OR agonist, is also considered to involve both signaling pathways and as a balanced ligand is sometimes used as a reference molecule [18] and positive control to study in vivo effects of novel compounds, as it induces behavioral effects thought to be processed by both G protein- and β-arr2-dependent signaling pathways. On the other hand, fentanyl, a powerful synthetic opioid analgesic has been described to be a β-arr2-biased agonist and this profile has been suggested to be responsible for its narrow therapeutic window [43].

The idea of biased agonism in the field of μ-OR ligands has its roots in studies on transgenic mouse models lacking β-arr2. Experiments have shown that morphine administration in these mice was associated with attenuated side effects, including respiratory depression and acute constipation [15]. Moreover, β-arr2 knockout mice presented an increased and prolonged morphine-induced antinociception [14] and failed to develop tolerance [46]. When compared with controls, β-arr2 knockout animals presented morphine-induced hyperlocomotion; however, to the lesser extent that controls [47]. In these mice, the physical dependence on morphine, measured as naloxone-precipitated withdrawal symptoms following chronic morphine exposure was unchanged [46] or slightly attenuated only at a low dose of daily morphine infusion [48]. Together with the observation that prolonged treatment with morphine led to an increase in adenylyl cyclase activity, a cellular hallmark of dependence, in both β-arr2 knockout and wildtype mice [46], it can be assumed that β-arr2 has no or a very little effect on opioid dependence. On the other hand, β-arr2 knockout mice presented increased sensitivity to rewarding properties of morphine in the conditioned place preference paradigm, which was accompanied by a greater release of striatal dopamine [47]. Interestingly, a study by Raehal and Bohn [48] has shown that the inhibition of antinociceptive tolerance in β-arr2 knockout mice is morphine-specific, as a chronic infusion of oxycodone, methadone, and fentanyl led to the same degree of tolerance and physical dependence. Alternate research with a model of functional knockdown of spinal opioid receptors using antisense oligonucleotides targeting β-arr2 has revealed a remarkably slower development of tolerance to morphine-induced antinociception [49]. Moreover, the treatment with β-arr2 antisenses resulted also in delayed development of allodynia in the rat model of neuropathic pain, suggesting that both tolerance and allodynia may be dependent on β-arr2-mediated receptor desensitization [49]. Similar results were observed in the study in which β-arr2 small interfering RNA (siRNA) was intrathecally injected to rats receiving a continuous intrathecal infusion of morphine. In particular, rats which were cotreated with morphine and β-arr2 siRNA displayed potentiated antinociception, diminished tolerance, and reduced naloxone-induced withdrawal symptoms, when compared with those treated only with morphine [50]. Therefore, these results support the idea that β-arr2 may contribute to morphine tolerance and physical dependence.

Based on abovementioned studies, it was hypothesized that biased agonists, through their ability to preferentially act via the G protein pathway, may present a desirable profile of in vivo effects. Following this hypothesis, several μ-OR agonists biased toward the G protein have been developed and widely studied over the last years.

## Addictive properties of G protein-biased μ-OR agonists

Addictive properties of several G protein-biased μ-OR agonists were tested in preclinical research, however, not all physical and subjective aspects of addiction have been studied with every substance. Below, we present currently available data on the influence of most important compounds from this family on addictive behavior.

### TRV130 (oliceridine)

One of the first compounds characterized as a G protein-biased μ-OR agonist was TRV130 (oliceridine), developed by Trevena Inc., using high-throughput screening of chemical library [51]. It was described as an agonist acting via the μ-OR and activating the G protein to a similar extent as morphine, but with minimal recruitment of β-arr2 [51]. According to the clinical data, oliceridine induces an analgesic effect between 5 and 20 min after the administration and has a relatively short half-life of 1–3 h [52]. Preclinical studies in rodents have shown antinociceptive efficacy of TRV130 in tests measuring acute nociception and this effect was approximately fourfold more potent when compared with morphine [51, 53–56]. It also caused less gastrointestinal dysfunction and respiratory suppression [51], indicating that its safety profile appears to be better when compared with conventional opioids.

Addictive properties of TRV130 were studied in several models assessing different aspects of addiction-related behavior. Mori et al. [57] reported that treatment with TRV130 did not induce the rapid development of tolerance in neuropathic mice. Similarly, enhanced resistance of TRV130 to antinociceptive tolerance was observed in other studies [55, 56]. At the same time, naloxone treatment in mice chronically treated with TRV130 induced withdrawal jumps, showing that mice became physically dependent on this drug [56].

In terms of rewarding effects of TVR130, measured in the conditioned place preference paradigm, reports are inconsistent. The data provided by Manglik et al. [58] showed that TRV130 did not have rewarding properties at low dose. Liang et al. [56] also revealed that this compound did not induce conditioned place preference when tested at a similar low dose (evoking equianalgesic effect as the tested dose of morphine), whereas a higher dose had a rewarding property. Therefore, rewarding effects of TRV130 seems to be dose-dependent, but at analgesic dose it does not induce reward-associated behavior, making its abuse potential relatively low.

In another study, TRV130 was tested for discriminative stimulus effects and it turned out that it produced fentanyl-like stimulus effect; however, lower than expected [53]. On the other hand, TRV130 produced constipating and abuse-related effects measured in intracranial self-stimulation procedure, however only after repeated treatment conditions [55]. In another study, TRV130 was shown to act as a reinforcer, because it was self-administered by rats [54]. These observations suggesting the abuse-related potential of TRV130 were confirmed in human studies, as participants of clinical trials reported that treatment with this compound is associated with morphine-like subjective effects of “high” [59].

Therefore, studies indicate that TRV130, retains some addiction-like properties, regardless of its bias toward the G protein. However, to date, no tolerance following repeated treatment with TRV130 was proven and this property makes this drug unique among opioid analgesics acting via μ-OR.

So far, this is the only molecule from G protein-biased opioids family, tested in clinical trials [59–63]. It was recently approved by the U.S. Food and Drug Administration for the management of moderate to severe acute pain in adults [64].

### Kratom alkaloids

*Mitragyna speciosa* (kratom) is a Southeast Asia evergreen tree. Leaves of this tree or their extracts were traditionally chewed to relieve pain, treat cough or gastrointestinal infections as well as to increase mood or libido [65]. Natural products of kratom represent different core structures, including mitragynine, mitragynine pseudoindoxyl, 7-hydroxymitragynine, and MGM-9, which were shown to display a bias toward the G protein. However, effects of these compounds are mediated not only via μ-OR, as some of them may at the same time act as δ-OR antagonists or κ-OR agonists [66–68].

Váradi et al. [66] reported in vitro and in vivo analysis of mitragynine pseudoindoxyl and its derivatives action. These compounds produced potent antinociception and had limited opioid side effects, such as respiratory depression and negative gastrointestinal symptoms. Interestingly, repeated treatment with mitragynine pseudoindoxyl resulted in slower development of antinociceptive tolerance and abolished withdrawal symptoms when compared with morphine. A recent study by Wilson et al. [69] also points out to the abolished withdrawal symptoms following the chronic treatment with mitragynince, mitragynine pseudoindxyl as well as with kratom alkaloid extract. Moreover, no rewarding properties of this substance were observed in the conditioned place preference test. The aforementioned kratom alkaloid, 7-hydroxymitragynine was reported to induce potent antinociception, also when administered orally, and was less constipating than morphine [70, 71]. Different studies have shown that treatment with 7-hydroxymitragynine results in the development of antinociceptive tolerance and withdrawal symptoms, that were comparable to those induced by morphine [72] and that it retains some rewarding properties in conditioned place preference test [73]. Hemby et al. [74] assessed possible reinforcing effects of both 7-hydroxymitragynine and isolated mitragynine. They have shown that 7-hydroxymitragynine, but not mitragynine, substituted for morphine self‐administration in rats, indicating reinforcing properties of 7-hydroxymitragynine. Yue et al. [75] also point out to limited abuse liability of mitragynine in self-administration paradigm. On the other hand, mitragynine possessed the discriminative stimulus effect in drug discrimination test [76]. MGM-9, another derivative of mitragynine, induced antinociception and weaker inhibition of gastrointestinal transit than morphine. It causes less rewarding effects when compared with morphine, but its repeated administration resulted in antinociceptive tolerance [67].

In summary, the data obtained so far suggest that kratom alkaloids might present useful templates for designing novel opioids with a more desirable profile of side effects.

### Kurkinorin

Kurkinorin, an analog of natural product salvinorin A, was also reported to be a pure μ-OR agonist and not to recruit β-arr2 [77–79]. Crowley et al. [78] have demonstrated that it presents a favorable profile of in vivo effects, including centrally-mediated antinociception as well as reduced tolerance and attenuated rewarding properties in conditioned place preference paradigm when compared with morphine. A variety of kurkinorin analogs was recently synthesized and evaluated. One of them (compound 25, lacking basic nitrogen, and other ionizable groups) displayed G protein bias with limited tolerance, suggesting that chemical modification of μ-OR agonists derived from salvinorin A are a promising class of compounds with limited abuse potential [78].

### Carfentanil-amide opioids

This group of compounds consists of structurally related to fentanyl opioid agonists which retain G protein but lose β-arr2 signaling [80]. The lead molecule has been described as a mixed μ-OR agonist/partial δ-OR agonist and presented antinociceptive potency. Interestingly, it caused no signs of physical dependence or constipation. A few carfentanil‐amide opioids were tested by Gurtidge et al. [73]. They have shown that carfentanil‐amide opioids do not strongly engage β‐arr2 pathway and vary in terms of efficacy at μ-OR and δ-OR. To date, little is known about behavioral pharmacology of these compounds and their abuse potential, but available data suggest that mixed μ-OR/δ-OR agonists with G protein bias appear as a promising group of opioid drugs and should be deeply investigated.

### Bifunctional G protein-biased μ-OR agonists/neuropeptide FF antagonists, KGFF03 and KGFF09

KGFF03 and KGFF09 are hybrid compounds. Both of them were described as G protein-biased μ-OR agonists, but KGFF03 presented full agonistic and KGFF09 full antagonistic activity at neuropeptide FF receptor [81]. Chronic treatment with KGFF09 did not result in the development of tolerance and induced less physical dependence than control substances, indicating that bifunctional compounds constitute an interesting strategy to develop opioids with reduced addictive properties.

### PZM21

PZM21 was discovered in 2016 using structure-guided computational modeling and originally was described to be an agonist of the μ-OR, potently activating of G protein pathway with minimal β-arr2 recruitment. PZM21 was effective in diminishing an “affective” component of pain and had reduced side effects, including respiratory depression and constipation [58]. The authors have also reported that PZM21 does not enhance the animals locomotor activity and does not lead to the formation of conditioned place preference, suggesting that it does not affect reward-associated behavior. This compound was extensively studied but some of its effects described in the original publication were not confirmed. Hill et al. [82] showed that PZM21 causes suppression of respiration. Kudla et al. [83] proved the data that PZM21 does not have rewarding properties in conditioned place preference procedure, both at the low dose tested by Manglik et al. [58] and at the higher doses which were more effective in evoking thermal antinociception. Interestingly, the authors demonstrated antinociceptive activity of PZM21 at the level of spinal reflexes which lasted longer when compared with morphine. In line with Hill et al. [82], it was again shown that PZM21 induces the rapid development of tolerance to antinociception. Repeated treatment with a high dose of PZM21 resulted in withdrawal symptoms precipitated by naloxone. The authors showed that, at least at the tested doses, PZM21 does not act as a reinforcer and does not induce craving and drug-seeking behaviors upon abstinence. To summarize, abovementioned studies suggest that PZM21 in a different way affects subjective (motivational and rewarding) and physiological (tolerance and dependence) aspects of addictive behavior. Although it causes antinociceptive tolerance and withdrawal symptoms, no rewarding or reinforcing properties of PZM21 were reported. However, more recent data provide observations regarding effects of PZM21 in nonhuman primates [84]. According to this study, PZM21 produces comparable to oxycodone reinforcing effects in self-administration test. Thus, the results indicate that reinforcing properties of PZM21 are revealed under certain conditions, such as dose, schedule of reinforcement, and animal model used in the study.

In summary, PZM21 also possess some addictive properties. Its repeated administration results in the development of antinociceptive tolerance, what strongly limits its possible clinical efficacy and at high doses it may be physically addictive.

### Piperidine benzimidazoles (SR-compounds)

SR-compounds belong to a group of piperidine-based ligands. The whole series has been described by Schmid et al. [43] and some of the SR-compounds were reported to be G protein-biased and selective for the μ-OR. When compared with morphine, SR-compounds were longer present and detected at higher concentration in plasma and brain tissue [43]. To date, several studies regarding their behavioral effects have been published, with most focusing on SR-14968 and SR-17018 as the most promising members of SR-compounds family. Both SR-14968 and SR-17018 were reported to induce antinociceptive responses [43, 53, 85]. The ligands appear to cause less respiratory depression than conventional opioids and have a broader therapeutic window, allowing for antinociception in the absence of respiratory suppression [43]. As far as their addictive properties are considered, Grim et al. [85] have shown that SR-17018 does not induce hot plate antinociceptive tolerance when administered chronically. A recently published study by Pantouli et al. [86] was aimed to determine the effectiveness of SR-17018 in mouse models of pain. Under repeated dosing, SR-17018 developed tolerance in the tail immersion test, suggesting that tolerance to antinociceptive effects of SR-17018 may occur when tests measuring spinal reflexes are used. Interestingly, repeated treatment with SR-17018 in mice with inflammatory and neuropathic pain did not result in tolerance, indicating that this compound retrains efficacy in pain conditions. In the abovementioned study [85], it was demonstrated that SR-17018 induces physical dependence, as mice chronically treated with this agonist had symptoms of abstinence-induced withdrawal. On the other hand, study regarding possible abuse-related discriminative stimulus effects of SR-14968 has shown that this compound may have a better ratio to produce antinociception vs abuse-related effects when compared to unbiased opioid analgesics [53]. However, the state-of-the-art on physiological and subjective aspects of the addictive potential of SR-compounds remains poor. The initial results seem to be promising, but more research is needed to characterize the influence of SR-compounds on various aspects of addictive behavior.

In conclusion, novel μ-OR agonists biased toward the G protein were assessed in different behavioral settings, however, most obtained results are not conclusive (Table 2). Based on the available behavioral data, it is difficult to assume, as yet, which features of these compounds are responsible for their effects on addiction-related symptoms. Nevertheless, they constitute very interesting tools for studying mechanisms involved in the formation of addiction. Possibly the combination of biased signaling and receptor binding profile(s) will allow the development of fully nonaddictive opioid analgesics in the future.

**Table 2:** The summary of currently available data about the impact of G protein biased μ-opioid receptor agonists on physiological and subjective aspects of addictive behavior

Agonist	Physiological aspects of addiction	Subjective aspects of addiction
TRV130 (oliceridine)	Increased resistance to antinociceptive tolerance [55–57] Physical dependence resulting in withdrawal symptoms [56]	Lack of rewarding effects at low dose, reward-associated behavior at high dose measured in conditioned place preference test [56, 58] Moderate abuse-related effects in drug discrimination test [53] Abuse-related effects in intracranial self-stimulation after repeated administration, a weak effect following acute treatment [55] Reinforcing effects in self-administration procedure [54] Morphine-like “high” feelings (in humans) [59]
Kratom alkaloids		
Mitragynine pseudoindoxyl	Slower development of antinociceptive tolerance [66] Limited physical dependence measured by abolished withdrawal symptoms [66, 69]	Lack of rewarding properties in conditioned place preference test [66]
7-Hydroxymitragynine	Development of antinociceptive tolerance [72] Physical dependence measured by withdrawal symptoms [72]	Rewarding properties in conditioned place preference test [73] Reinforcing effects in self-administration paradigm [74]
Mitragynine	Diminished physical dependence measured by withdrawal symptoms [69]	Limited reinforcing effects in self-administration paradigm [74, 75] Discriminative stimulus properties in drug discrimination test [76]
MGM-9	Development of tolerance to antinociception [67]	Reduced hyperlocomotion [67] Abolished rewarding effects in conditioned place preference test [67]
Kurkinorin	Reduced development of tolerance to antinociception [78]	Diminished rewarding effects in conditioned place preference test [78]
Carfentanil-amide opioids	Possibly abolished physical dependence and withdrawal symptoms [80]	
KGFF09	Increased resistance to antinociceptive tolerance [81] Reduced physical dependence measured by withdrawal symptoms [81]	
PZM21	Development of antinociceptive tolerance [82, 83] Physical dependence resulting in withdrawal symptoms, but only at high dose [83]	Lack of rewarding effects in conditioned place preference test [58, 83] No enhancement of locomotor activity and no locomotor sensitization [58, 83] No reinforcing effects in self-administration study in rodents and no drug-seeking behaviors [83] Reinforcing effects in self-administration paradigm in primates [84]
Piperidine benzimidazoles (SR-compounds)		
SR-14968		Attenuated discriminative stimulus properties in drug discrimination test [53]
SR-17018	Increased resistance to antinociceptive tolerance in hot plate test [85], development of tolerance in tail immersion test but no tolerance after repeated treatment in mice with inflammatory and neuropathic pain [86] Abstinence-induced withdrawal symptoms [85]	

Data are derived from rodent studies unless otherwise noted

## The influence of G protein-biased μ-OR agonists on symptoms of addictive behavior evoked caused by conventional opioid drugs

Apart from described above basic studies aimed at assessing the addictive potential of G protein-biased μ-OR agonists, another interesting area of research is focused on the evaluation of these compounds in terms of their potential use in the pharmacotherapy of opioid addiction. Currently, the pharmacotherapy of opioid use disorder is focused on opioid replacement therapy that involves replacing a highly addictive opioid with a longer acting, but less euphoric drug (such as methadone or buprenorphine) [87]. However, novel compounds with a similar profile are still desired. Below we discuss the current data on the possible use of biased μ-OR agonists in the treatment of opioid addiction-like behavior.

### TRV130 (oliceridine)

Mori et al. [57] determined the influence of TRV130 on tolerance to the antihyperalgesic effect of fentanyl (an opioid presenting profound β-arr2 recruitment) in neuropathic mice. The results revealed that mice additionally treated with TRV130 did not develop rapid tolerance, as in the case when they were treated with fentanyl alone. In the same study, the authors showed that TRV130 enhanced the antinociceptive effect of fentanyl. Therefore, this study provides evidence that the combination of G protein-biased (TRV130) and β-arr2-biased (fentanyl) agonists of μ-OR may be useful in the exertion of analgesia without the rapid development of tolerance.

Another study was aimed to determine the influence of TRV130 on symptoms of oxycodone oxycodone seeking and taking during the abstinence period in comparison to buprenorphine [88]. The obtained results revealed that TRV130 attenuated oxycodone seeking and taking during abstinence, interestingly—in a sex-dependent manner. Moreover, in both males and females, treatment with TRV130 prevented oxycodone-induced brain hypoxia. Taken together, the authors suggest that TRV130 may be potentially useful as a maintenance medication, as it may be safer than methadone and more effective than buprenorphine.

### Kratom alkaloids

Mitragynine (kratom) was historically used as an opium substitute in East Asia countries [89, 90]. It was also reported by online survey participants to decrease opioid use, withdrawal and craving [91]. Another report indicates that mitragynine and mitragynine pseudoindoxyl diminished precipitated withdrawal in morphine-dependent mice [69]. In the study performed by Yue et al. [75] rats trained to self-administer heroin were pretreated with mitragynine and, in effect, presented decreased response rates maintained by heroin. Interestingly, this effect seemed to be selective for heroin, as mitragynine had a very little effect on self-administration of methamphetamine. What is more, Hemby et al. [74] have shown that mitragynine reduced morphine intake in self-administration procedure. Another recent study indicated that kratom alkaloids can also reduce alcohol intake in mice [73]. Taken together, both human reports and preclinical research suggest that kratom alkaloids, especially pure isolated kratom, may successfully diminish various aspects of opioid addiction.

### PZM21

Kudla et al. [83] tested the influence of PZM21 on morphine-induced antinociceptive tolerance, locomotor sensitization and conditioned place preference. The results showed that PZM21 had no effect on the development of morphine tolerance as well as hyperlocomotion and its sensitization. However, PZM21 prevented the formation of conditioned response to morphine, indicating that it may suppress rewarding properties of morphine.

### Piperidine benzimidazoles (SR-compounds)

To date, only SR-17018 was tested in terms of its impact on some aspects of addictive behavior [85]. The study revealed that substitution with SR-17018 in mice tolerant to morphine was able to restore morphine potency and efficacy in hot plate test. Moreover, it prevented the onset of morphine withdrawal. However, recent data show that SR-17018 did not reverse morphine tolerance in tail flick assay [86]. These results suggest that SR-17018 may efficiently attenuate some aspects of opioid addictive behavior, but not under all circumstances.

To sum up, data collected so far suggest that G protein-biased μ-OR agonists may modulate behavioral manifestations of opioid addiction (Table 3). TRV130, SR-17018, and kratom alkaloids were shown to attenuate tolerance and withdrawal symptoms to other opioid drugs—therefore may be potentially used in combination therapy to prolong or restore the efficacy of conventional opioids as well as in maintenance treatment to reduce withdrawal. On the other hand, PZM21 had a positive influence on the subjective aspect of opioid addiction, as it sup pressed the expression of morphine reward, indicating that it may be considered as a novel compound in combination therapy, able to diminish unwanted effects of “high” in the course of opioid pharmacotherapy.

**Table 3:** Summary of the described effects of G protein biased μ-opioid receptor agonists on physiological and subjective aspects of addiction to other opioid drugs

Agonist	Physiological aspects of addiction	Subjective aspects of addiction
TRV130 (oliceridine)	Attenuation of antinociceptive tolerance to fentanyl [57]	Attenuation of oxycodone seeking and taking during abstinence (in a sex-dependent manner) [88]
Kratom alkaloids		
Mitragynine pseudoindoxyl	Attenuation of withdrawal symptoms in morphine-dependent mice [69]	
Mitragynine	Used as opium substitute by Asian and reported to decrease withdrawal symptoms in humans [89–92] Attenuation of withdrawal symptoms in morphine-dependent mice [69]	Reported to reduce craving by online surveyed [91] Attenuation of reinforcing properties of heroin [75] and morphine [74]
PZM21	No effects on the development of antinociceptive tolerance to morphine [83]	Attenuation of rewarding properties of morphine in conditioned place preference test [83] No effects on morphine-induced locomotor activity and sensitization [83]
Piperidine benzimidazoles (SR-compounds)		
SR-17018	Reversion of antinociceptive tolerance to morphine in hot plate [85], but not in tail flick test [86] Prevention of morphine withdrawal [85]	

Data are derived from rodent studies unless otherwise noted

## Biased signaling or other factors? Which pharmacological properties of novel compounds may be responsible for their in vivo effects?

### Re-evaluation of the role of β-arr2 in mediating of opioid side effects

There is currently no doubt that the development of G protein-biased μ-OR agonists made possible a better insight into functional roles of G protein- and β-arr2-dependent signaling pathways in mediating of therapeutic and adverse effects of opioid drugs. However, a better understanding of the opioid system pharmacology does not easily translate into developing new safe and nonaddictive opioids and, in that regard, G protein-biased μ-OR agonists have not, so far, fulfilled hopes of clinically useful compounds.

Although the hypothesis that β-arr2 is responsible for undesirable opioid effects was the trigger for novel drugs development, latest reports indicate that dependence between specific intracellular pathways involved and drug effects in vivo is much more complex and still requires comprehensive research. An original mouse model with β-arr2 knockout has the serious drawback of causing the global ablation of β-arr2, thus it is not restricted to the μ-OR. A more recent study by Kliewer et al. [93] describes the effects of opioids administration in genetically modified mice, expressing modified μ-OR preventing phosphorylation and binding of β-arr2 (11S/T-A μ-OR knock-in mice). The obtained data indicate that, when μ-OR were unable to recruit β-arr2, mice displayed unchanged respiratory depression and constipation following treatment with morphine and fentanyl. Somatic signs of withdrawal were also retained in these mice, regardless of no β-arr2 binding to μ-OR. Both mutant and control animals exhibited morphine- and fentanyl-induced hyperlocomotion and reward-associated memory in a conditioned place preference test. Interestingly, in line with the results obtained using β-arr2 knockout mice, opioid-induced antinociception was enhanced and tolerance was attenuated in mice unable to bind β-arr2 to μ-OR. Therefore, this study does not provide evidence that the severity of adverse effects associated with morphine and fentanyl administration is dependent on μ-OR recruitment of β-arr2, while it supports the hypothesis that lack of β-arr2 binding may contribute to the potentiation of opioid antinociceptive efficacy and tolerance diminishment.

Another study was aimed to re-examine the role of β-arr2 in respiratory depression and constipation using the same β‐arr2 knockout mouse line as in the initial investigations. However, the effects of morphine and fentanyl on suppression of respiration and acute constipation was independently assessed in three laboratories and all of them failed to reproduce the previous results, as respiratory depression and constipation were observed in both genotypes [94]. Therefore, novel data call into question the causal dependence between the β-arr2 engagement and safety profile of opioids. There is substantial evidence suggesting that undesirable opioid effects, including respiratory depression and constipation, are not directly connected with β-arr2 signaling. An elegant summary of this topic was recently provided by Gillis et al. [16] and Table 4 presents the currently available data on the morphine effects in various models with disrupted β-arr2 functions.

**Table 4:** Morphine effects in animals with disrupted β-arr2 functions

Effects on morphine-induced:	β-arr2 knockout mice^a^ [14, 15, 46, 47]	β-arr2 antisense-treated mice [49]	β-arr2 siRNA-treated mice [50]	11S/T-A μ-OR knock-in mice [93]	β-arr2 knockout mice^b^ [94]
Respiratory depression	↓	–	–	↔ /↑	↔
Constipation	↓	–	–	↔	↔
Antinociception	↑	–	↑	↑	–
Tolerance to antinociception	↓	↓	↓	↓	–
Physical dependence (withdrawal symptoms)	↔	–	↓	↔	–
Reward sensitivity	↑	–	–	↔	–
Hyperlocomotion	↓	–	–	↔	–

↓ decrease, ↑ increase, ↔ no changes, – no data

^a^Initial studies

^b^Replication studies

To sum up the interplay between the β-arr2 signaling and addictive symptoms, data obtained so far, have brought up inconclusive outcomes. As far as physiological aspects of addiction are considered, studies using both models of genetically modified animals and research using antisense treatment suggest that β-arr2 is, at least to some degree, involved in the formation of antinociceptive tolerance. However, this hypothesis may not necessarily be precisely translated into the action of the biased ligands, as different G protein-biased agonists of μ-OR, had varying effects on tolerance progress, e.g., TRV130 turned out to be resistant to tolerance, while treatment with PZM21 was associated with rapid tolerance development. Opioid tolerance appears to be rather a ligand-specific feature and may involve both β-arr2-dependent and independent mechanisms. The potential of a given agonist to produce tolerance is also connected with its efficacy, since it was shown that those with higher efficacy require lower receptor reserves to preserve analgesic properties and in turn, cause slower development of tolerance [95–97]. Given the abovementioned arguments, it can be assumed that β-arr2-dependent processes may, to some extent, contribute to the tolerance development, but it does not seem that avoiding β-arr2 recruitment will be sufficient to prevent it. The importance of β-arr2 signaling in the physical dependence and occurring of the withdrawal symptoms were studied using several approaches. Evidence from transgenic mouse models and experiments in which treatments disrupting the β-arr2 functions were used provided ambiguous results. At the same time, the potential of G protein-biased μ-OR agonists to evoke physical dependence seems to be rather small, because they were not reported to induce severe withdrawal symptoms. Therefore, it again appears that avoiding of β-arr2 recruitment may have a small impact on the development of physical dependence on some of the opioid drugs; however, presumably other pharmacological properties of a given molecule are in this context more significant.

Several studies were aimed to investigate the possible interdependence between β-arr2 functions and addictive behaviors related to reward and reinforcement. Initial research in transgenic mice suggested that β-arr2 may be partly responsible for typical running behavior associated with morphine administration. Another research has shown that morphine-induced hyperlocomotion depends on G protein-coupled receptor kinase 6 (GRK6), as GRK6 knockout mice presented greater locomotor activity following morphine treatment and overexpression of GRK6 in cell cultures was associated with facilitated morphine-induced β-arr2 recruitment and receptor internalization [98]. Morphine-induced hyperlocomotion in mice is considered to be a dopamine-related phenomena [99]. It was shown that morphine-induced enhancement of locomotor activity requires β-arr2 in a D1 receptor-dependent manner [100]. In accordance, currently available data indicate that μ-OR agonists with G protein bias do not enhance an acute locomotor activity of the rodents.

On the other hand, β-arr2 knockout mice displayed increased sensitivity to the rewarding properties of morphine in the conditioned place preference paradigm [47]. In contrast to the morphine-evoked hyperlocomotion, reward-associated behavior is not believed to be mediated by a D1 receptor-dependent β-arr2/pERK cascade [100] and is independent on dopamine signaling, at least when the reward is measured in conditioned place preference paradigm [101, 102]. Furthermore, loss of morphine reward was observed in mice with G protein-coupled receptor kinase 5 (GRK5) knockout, as GRK5 may serve a scaffolding function facilitating signaling through the morphine-activated μ-OR [103]. Thus, there are some important differences in both molecular and neurotransmitters systems that may underlie the separation of the mechanisms responsible for morphine-induced locomotor activity and rewarding effects. Nevertheless, G protein-biased μ-OR agonists were proposed as a class of drugs with possibly abolished rewarding effects [58]. Indeed, this hypothesis was supported in several studies, since TRV130, PZM21, and mitragynine were reported not to induce conditioned place preference, at least at most commonly used doses. However, the question regarding the actual mechanisms responsible for those observations remains open.

Based on available data, it is difficult to anticipate the role of β-arr2 in other subjective aspects of opioid addiction, such as drug-induced reinforcement. It is difficult to make a comprehensive conclusion based on transgenic studies and pharmacological research using G protein-biased compounds. Reinforcing effects of G protein-biased μ-OR agonists are also a matter of debate and probably are dependent on the dose and specific methodological issues, as the literature provides us with both positive and negative results regarding the effects of the compounds on operant behavior. It should be also noted, that investigation of subjective effects of addiction in rodents is fraught with some risk and methodological weaknesses and such results do not have to translate into similar observations in primates, including humans, for example, PZM21 was not self-administered by rodents [83], while an opposite effect was observed in monkeys [84].

To summarize, recent advancements in our understanding of the interplay between GPCR signaling pathways and addictive properties of μ-OR agonists do not allow us to unequivocally state that separation of certain pathways is entirely possible and has relevance to better in vivo profile of opioid compounds. The attribution of all undesirable opioid effects to β-arr2 signaling appears to be an oversimplification of intracellular signaling pathways, as the development of different addiction symptoms involves both β-arr2-dependent and independent mechanisms. The complexity of μ-OR signaling cannot be reduced to the two pathways but requires taking into account that β-arr2, as a scaffolding protein, regulates the variety of signaling cascades that, in turn, differently affect mechanisms underlying addictive behavior [98, 103, 104]. Diminished addictive symptoms of certain agonists reported in some studies are rather a ligand-specific trait and may be considered as outcomes of the combination of G protein bias and other pharmacological properties.

## Low intrinsic efficacy and partial agonism of novel compounds as possible causes of their behavioral profiles

As described in the previous section, the current status of compounds proposed to be G protein-biased μ-OR agonists as nonaddictive and safe opioid analgesics is questionable. In this context, it is extremely important to pay attention to their other pharmacological properties, not only to G protein bias. A significant factor that may have relevance to the behavioral effects of the abovementioned μ-OR agonists is their lower intrinsic G protein efficacy, referring to the relative ability of the drug–receptor complex to produce a functional response. Opioid agonists display different levels of intrinsic efficacy, therefore can be ranked in terms of this attribute. Fentanyl and hydromorphone are full μ-OR agonists with high efficacy, morphine has a slightly lower intrinsic activity, while buprenorphine, a partial μ-OR agonist, displays a very low efficacy, but produces reasonable analgesia [105, 106]. What is important, partial opioid agonists are usually considered to be safer than full agonists because of a higher therapeutic index (analgesia versus respiratory depression) and consequently are less likely to cause fatal respiratory suppression [107]. It should be noted that the bias factor does not correspond to the efficacy of a given agonist. A level of efficacy (full/partial agonistic activity of a ligand) is associated with the extent to which an agonist stimulates certain pathway in cellular assays. To determine the bias factor, the relation between the levels of recruitment of both pathways must be assessed. Thus, an agonist should be considered in terms of its bias profile and intrinsic efficacy separately. Those properties may independently contribute to the safety profile of a given ligand.

To date, at least a few of these compounds were shown to act as partial μ-OR agonists. Hill et al. [82] showed that PZM21 induces suppression of respiration and behave as a low efficacy partial agonist of μ-OR. This property of PZM21 became proved by Yudih and Rohacs [108] using patch–clamp electrophysiology and Ca^2+^ imaging. Interestingly, the same observation was also attributed to TRV130. Behavioral characterization of PZM21 [83] also suggests that PZM21 induces in vivo effects typical for partial μ-OR agonists, such as ceiling effect in antinociceptive tests and inhibitory influence on morphine reward (partial agonists of μ-OR, including buprenorphine and nalbuphine, are useful in opioid addiction therapy [109–112]). In terms of antinociceptive tolerance, lower tolerance was usually attributed to high efficacy μ-OR agonists [95, 113, 114], thus in this context, the described tolerance to PZM21 could be explained as a consequence of its low efficacy profile.

Another study revealed that low intrinsic efficacy should be assigned to TRV130, PZM21, and SR-17018 [115]. Importantly, the authors have demonstrated that the therapeutic window (antinociception in the absence of respiratory depression) of opioid ligands is inversely correlated with their intrinsic efficacy. In this study, bias factor did not correlate with increasing therapeutic window, suggesting that low intrinsic efficacy for G protein activation, rather than G protein bias, is crucial for opioid safety profile. No operational analysis data on the correlation between intrinsic efficacy/bias factor and addictive potential of opioid compounds are available at this moment. Nevertheless, experimental results, e.g., [83, 85], support the hypothesis that, in case of SR-17018 and PZM21, some favorable effects of these compounds (i.e., lack of antinociceptive tolerance following the treatment with SR-17018 and no rewarding effects of PZM21) may be at least partly connected with their low intrinsic activity. Thus, whereas biased agonism is one hypothesis that may be important for the development of opioid drugs with reduced side effects, more recent analyses indicate that low G protein efficacy may have greater contribution to the favorable safety profile of new opioids. A comprehensive review was provided by Azevedo Neto et al. [116]. It also should be noted that the relationship between the G protein bias and addictive potential of a ligand is difficult to assess due to methodological issues connected with bias estimation. In fact, in some cases, the status of novel compounds as G protein-biased has been called into question and the extent of bias signaling turned to be limited [43, 58, 82, 117]. Moreover, across studies, determination of bias factors was based on different methodological assays, leading to inconsistencies in the obtained data [118]. Importantly, G protein assays may be insensitive to variations in agonists efficacy, which can explain the initial description of some compounds as highly efficacious for the activation of G protein [16]. Several studies and comprehensive reviews on this matter have been published recently [18, 117–119].

### Other approaches to search for safe and nonaddictive opioid drugs

Although biased signaling at μ-OR has received the most attention in searching for opioids devoid of undesirable effects, there are also other approaches worth mentioning. G protein-biased agonists of κ-OR are a promising group of opioid compounds, as it is hypothesized that such drugs can diminish pain and itch, while having fewer side effects, including decreased anhedonia, which is typical for unbiased κ-OR agonists [120]. On the other hand, G protein-biased δ-OR agonists are an interesting alternative for antihyperalgesic and antidepressant treatments with abolished tolerance, reward, and proconvulsant effects [121]. Another concept is the development of opioid ligands acting only on the periphery, such as pH-dependent agonist, which was also reported not to induce reward-associated behavior [122, 123]. A large group of intensively studied compounds are also different hybrid compounds with mixed agonistic/antagonistic activity at either opioid or nonopioid receptors. Searching for compounds with complex receptor binding profiles allow synergy of therapeutic action and avoidance of some undesirable effects, e.g., agonism of μ-OR and δ-OR antagonism lead to antinociception with no tolerance [12], while bifunctional hybrids composed of opioid receptor agonist and MC4 receptor antagonist delay the development of tolerance in rats with neuropathic pain [124]. It is also worth mentioning that µ-δ-OR heterodimers may allow inducing antinociception with lower tolerance under repeated dosing [125, 126].

Among last years, various alternatives to discover efficacious, safe, and nonaddictive opioids were assessed; however, the problem has not yet been resolved. Surely, advances in opioid pharmacology resulted in changes in old doctrine, stating that high affinity and high efficacy μ-OR agonists would be the best analgesic agents. For now, the opioid drugs discovery is moving towards searching for molecules with more complex pharmacology, selectively acting on specific signaling pathways or tissues and interacting with different receptors and even neurotransmitter systems.

## Conclusions

The hypothesis that the preferential activation of G protein- over β-arr2-dependent signaling pathway can reduce or remove the opioid side effects has dominated the field of opioid drugs discovery in recent years. It should be noted that G protein-biased opioids are not a homogenous group—they differ in terms of bias factor, intrinsic activity or receptors selectivity. Currently available data provided by various research groups suggest that putative G protein-biased agonists of μ-OR exhibit a low agonistic efficacy and some desirable aspects of their action may be attributed not only to their G protein bias but also to this pharmacological property.

Development of nonaddictive opioid remains a challenge, but thanks to the latest scientific achievements in this field, a list of properties that such a hypothetical compound should possess is being created; functional selectivity appears to be one of them. Although, as shown in this review, many aspects of the addictive potential of novel μ-OR agonists remain controversial, compounds developed as the result of presented studies, in some cases represent an attractive alternative to currently used conventional opioids. Some of them exhibit favorable properties, such as prolonged antinociceptive action, delayed tolerance development or ability to inhibit addiction symptoms to other opioid drugs. It makes them inspiring candidates for further research focused on a better understanding of functional selectivity and novel G protein-biased opioids design and development.

## Abbreviations

δ-OR: δ-Opioid receptor
μ-OR: μ-Opioid receptor
κ-OR: κ-Opioid receptor
β-arr2: β-Arrestin2
cAMP: Cyclic AMP
GPCRs: G protein coupled receptors
GRK5/GRK6: G protein-coupled receptor kinase 5/G protein-coupled receptor kinase 6
OUD: Opioid use disorder
siRNA: Small interfering RNA
